# Densely Packed and Highly Ordered Carbon Flower Particles for High Volumetric Performance

**DOI:** 10.1002/smsc.202000067

**Published:** 2021-03-14

**Authors:** Huaxin Gong, Shucheng Chen, Rui Ning, Ting-Hsiang Chang, Jeffrey B.-H. Tok, Zhenan Bao

**Affiliations:** ^1^ Department of Chemical Engineering Stanford University Stanford CA 94305 USA; ^2^ Department of Materials Science and Engineering Stanford University Stanford CA 94305 USA

**Keywords:** activated carbon, carbon monoliths, close packing, self-assembly of carbon flowers, volumetric performances

## Abstract

Carbon materials with high specific surface areas are ideal support materials for many applications. However, high specific surface area and large pore volume usually render them with low bulk density, which is undesirable for applications aiming at high volumetric performance. Low bulk density stems from large interparticle‐free volume caused by inefficient random packing within the materials. Herein, a simple synthesis and assembly method is reported to afford dense carbon pellets with both high specific surface area and high bulk density, obtained from the ordered packing of low polydispersity carbon flower particles. The densely packed carbon flower particles exhibit similar specific surface area to their pressed powder analogs, while exhibiting a 66–84% increase in bulk density (0.815 g cm^−3^), and an ultrahigh volumetric surface area (1081 m^2^ cm^−3^). The advantages of our materials are demonstrated by supercapacitors, which achieve a high volumetric capacitance of up to 153 F cm^−3^. The results reinforce the importance of controlling particle size and shape for porous materials to reduce their bulk volume. The developed materials possessing high volumetric surface area will be useful for many applications, such as gas storage, supercapacitors, and batteries.

## Graphical abstract

A strategy to synthesize high‐bulk‐density carbon materials by self‐assembling carbon flower particles into ordered arrays is reported. The densely packed carbon flowers demonstrated similar gravimetric surface areas to their powder analogs, while exhibiting up to 84% improvement in bulk density and a high volumetric surface area (1081 m^2^ cm^−3^). These materials are promising for many energy storage related applications.

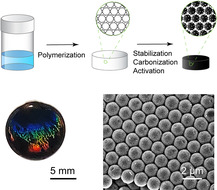

## 1 Introduction

Unprecedented demand for increasing power in our fast‐growing electronics and vehicle technologies requires new materials capable of higher energy and gas storage capability. Porous carbons are ideal materials for the aforementioned purposes due to their high specific surface area, good conductivity, and chemical stability. Previous studies have shown that porous carbon materials have excellent gravimetric performances in many energy and gas storage‐related applications such as batteries,^[^ ^1^, ^2^, ^3^ ^]^ gas storage and separation,^[^ ^4^, ^5^ ^]^ and supercapacitors.^[^ ^6^, ^7^ ^]^ However, their volumetric performance needs further improvement for practical applications in current highly compact electronic devices and vehicles.^[^ ^8^, ^9^ ^]^ Porous carbon with high specific surface area and large pore volume usually has low bulk density, resulting in increased bulk material volume and poor volumetric performance. For example, even though lithium‐sulfur batteries have shown excellent specific energy of 350–400 Wh kg^−1^ at the cell level, which is ≈2× of conventional Li‐ion batteries, their energy density (Wh L^−1^) is much lower compared with Li‐ion batteries at the device level.^[^ ^10^ ^]^ One main reason is the usage of low‐bulk‐density porous carbon as the host material and conducting agent in the batteries.

The low bulk density of porous carbon materials stems mainly from their irregular particle shape and size. Most of the porous carbon materials that are powdered comprise distinct carbon particles. Random packing of these carbon particles leads to large interparticle free volume, thus resulting in oversized pores in the material. These large pores occupy a large proportion of the material volume, which then reduce the materials overall bulk density. Unfortunately, they do not contribute much to enhance the overall performance. In addition, this large interparticle‐free volume cannot be left as voids and needs to be “filled‐up” with extra electrolytes to form a continuous conductive network in many electrochemical energy storage‐related applications. The additional electrolyte subsequently increases the total weight of the material, which in turn lowers the overall gravimetric performance. Therefore, it is essential to reduce the interparticle free volume of porous carbon materials to achieve high bulk density and ideal performance.

To address this issue, different strategies were reported to increase the bulk densities of porous carbon materials, such as: mechanical compression,^[^ ^11^, ^12^, ^13^ ^]^ vacuum filtration,^[^ ^7^ ^]^ capillary densification,^[^ ^14^, ^15^, ^16^ ^]^ and filling with active components.^[^ ^17^ ^]^ Among these techniques, a common approach is to use mechanical compression to reduce interparticle‐free volume. By compressing the mixture of carbon particles and binder in a mold, dense monoliths can be fabricated. However, this process could damage the structure of the original material.^[^ ^13^ ^]^ In addition, the extra weight from the binder would result in decreased gravimetric performance. Another strategy to remove the large interparticle‐free volume is “capillary densification.” This method involves porous carbon synthesis, the gelation of active materials, and capillary evaporation‐induced drying (CEID). However, this process is typically tedious and the materials usually suffer from insufficient mechanical strength.^[^ ^14^, ^15^, ^16^ ^]^

Herein, we report a strategy to effectively reduce interparticle‐free volume by self‐assembling carbon particles into an ordered array to improve the bulk density. First, our carbon particles are highly uniform flower‐shaped carbon microspheres derived from polyacrylonitrile particles we had recently developed.^[^ ^18^ ^]^ Second, our flower carbon microspheres were observed to show promising performances in many areas, such as flexible electronics, methane storage, and electrocatalysis.^[^ ^19^, ^20^, ^21^ ^]^ By organizing randomly packed carbon flowers into orderly and densely packed carbon structures, the interparticle‐free volume could be considerably reduced without causing damage to the original carbon structure. Hence, with both high bulk density and high gravimetric surface area obtained, a high volumetric surface area can then be achieved. Indeed, our fabricated densely packed carbon flowers demonstrated similar surface areas to their powder analogs (1326 m^2^ g^−1^), while exhibiting a 66–84% improvement of bulk density (0.815 g cm^−3^) as compared with randomly packed carbon flower particles. Our obtained materials also showed a high volumetric surface area (1081 m^2^ cm^−3^). In the supercapacitor application, our ordered carbon flowers showed clear advantages over the randomly packed carbon flowers, and achieved a high volumetric capacitance of 153 F cm^−3^. These obtained results reinforce our hypothesis that controlling particle packing in porous materials is able to effectively reduce the bulk volume of the material. We anticipate that our developed materials with high volumetric performance to be promising for a number of applications, such as gas storage, supercapacitors, and batteries.

## 2 Results and Discussions

### 2.1 Synthesis of Densely Packed Carbon Flower Pellets

**Figure 1:**
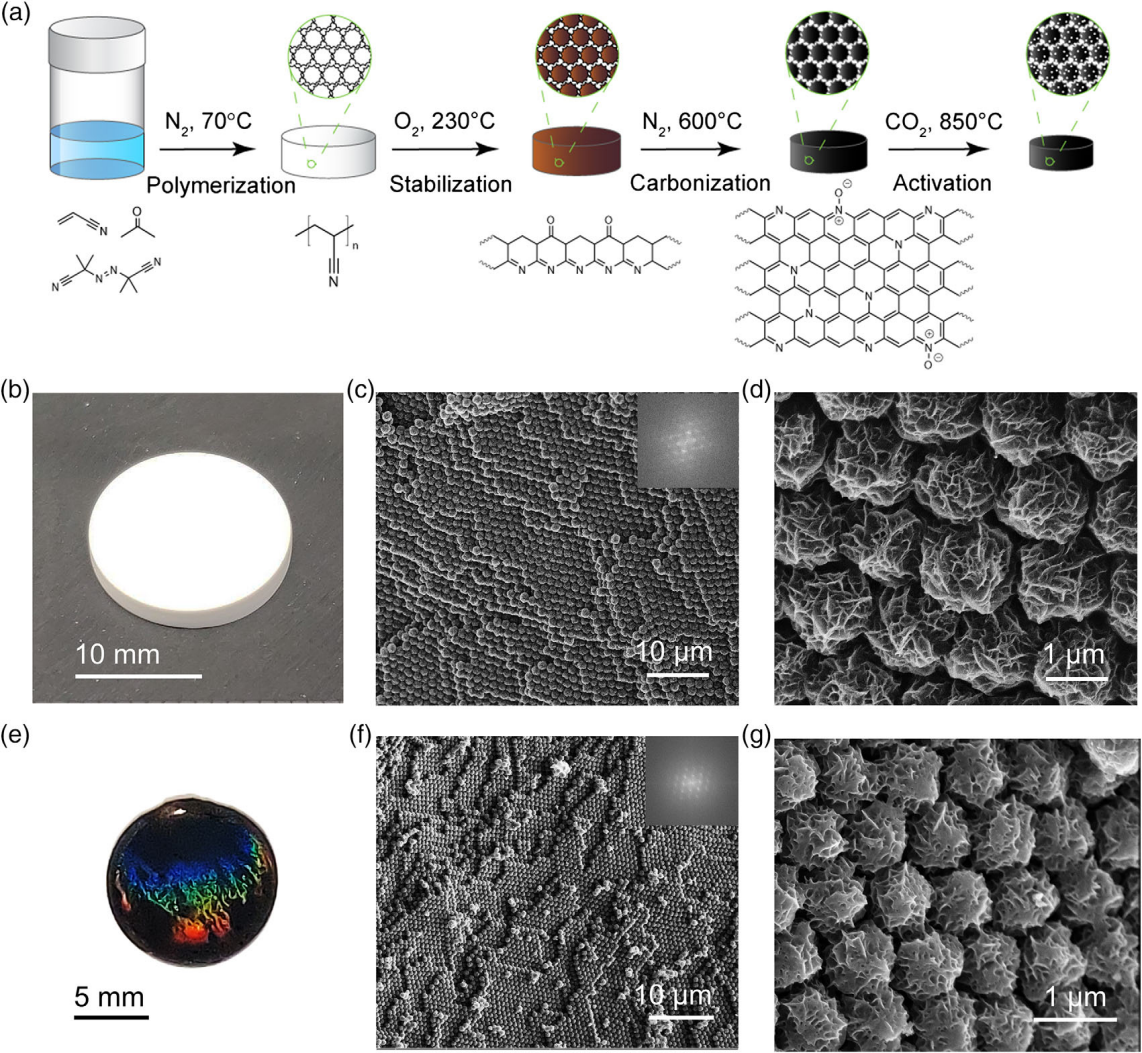
a) Synthetic process of activated carbon flower pellet assemblies (ACFP‐SAs). b) A photo of a polyacrylonitrile flower pellet (PANFP). c,d) SEM images of a PANFP with a Fast Fourier Transform (FFT) image on the top‐right corner of (c). e) A photo of an ACFP‐SA showing opal‐like colorful patterns. f,g) SEM images of an ACFP, with an FFT image on the top‐right corner of (f).

**Figure** **1**a shows the synthetic approach to obtain densely packed carbon flower arrays. We previously reported the synthesis of carbon flower particles and methods to control their shapes and sizes.^[^ ^18^ ^]^ In this study, we found that a higher concentration solution and longer reaction time (>3 h to overnight) can lead to the formation of ordered and densely packed carbon flower pellets (CFPs) at end of the reaction. Shapes of the pellets could be easily tuned using different containers (Figure S1, Supporting Information). Specifically, acrylonitrile was polymerized in acetone with 2,2′‐azobis(2‐methylpropionitrile) (AIBN) as the initiator at 70 °. Freestanding polyacrylonitrile pellets (PANFPs) consisting of ordered polyacrylonitrile flower (PANF) arrays were then achieved. The following stabilization and carbonization processes converted the polymer pellets into CFPs with retained physical structures. Last, activated‐carbon flower pellets (ACFPs) with increased specific surface area were afforded after a 9 h CO_2_ activation at 850 °C. During the polymerization process, polymer‐solvent interaction produced narrowly dispersed PANF as governed by Hansen Solubility Parameters,^[^ ^18^ ^]^ and the longer reaction time provided enough time for PANF particles to self‐assemble into ordered arrays to form a freestanding pellet. Figure 1b shows the freestanding PANF pellet, and Figure 1c,d shows the representative scanning electron microscope (SEM) images at different scales. The PANF synthesis and assembly processes can be accomplished in a one‐pot reaction within only a 3 h duration. This synthetic approach is advantageous over usual synthetic processes of ordered sphere arrays, which involves tedious synthesis steps of monodispersed spheres, surface grafting, redispersing, and assembly during controlled drying.^[^ ^22^, ^23^, ^24^ ^]^

**Figure 2:**
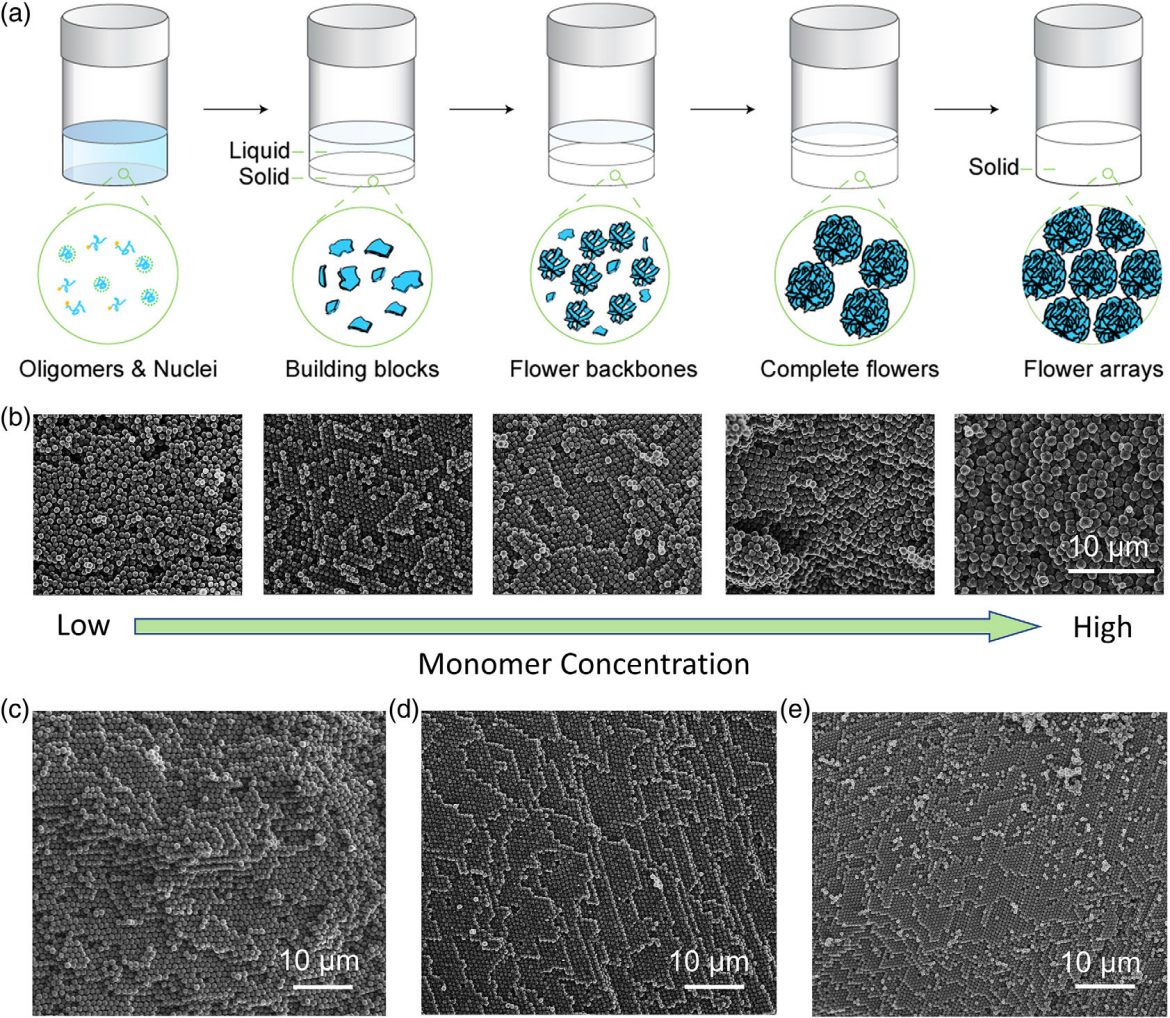
a) A schematic diagram of the PANFP formation process. b) Influence of monomer concentrations on the packing observed by SEM images. (from left to right, 36, 44, 50, 57, and 66 vol%, respectively). All images have the same scale. c–e) Ordered packing observed by SEM could be achieved with many selected solvents in similar monomer concentration range (from left to right, acetone, THF, and pyridine, respectively).

After polymerization, PANF could be easily converted to carbon materials without suffering structural collapse. The main reason is because during its stabilization step in air, polyacrylonitrile forms a ladder polymer structure from a cyclization reaction at around 230 °C,^[^ ^18^ ^]^ enabling it to withstand subsequent high‐temperature thermal treatment and be converted into carbon without structural collapsing. Its well‐preserved morphology after carbonization is shown in **Figure** **2**c and Figure S2, Supporting Information. Furthermore, we observed that its structures remained well preserved, i.e., from single flower particles to ordered arrays, even after a 9 h CO_2_ activation, as shown in Figure 1f,g. The densely packed arrays were clearly observed throughout the freestanding pellet, as shown in Figure S3, Supporting Information. The diameter for some particles was observed to be <700 nm after activation and showed “colorful” opal‐like patterns (Figure 1e and Figure S4, Supporting Information). This is caused by optical interference and diffraction effects from the periodic structures, indicating that the ordered structures exist throughout the freestanding pellet up to the centimeter scale. Typically, ordered sphere arrays may require a long duration (from months to years) to form centimeter‐scale freestanding ordered structure.^[^ ^25^ ^]^ Furthermore, our freestanding pellets have outstanding mechanical strength without the need to add any binders. As shown in Video 1 and Figure S5a, Supporting Information, a pressure as high as 127 kPa can be applied without collapsing the pellet. Their excellent mechanical strength was retained even after CO_2_ activation (Video 2 and Figure S5b, Supporting Information), considering CO_2_ activation usually etches carbon materials to render them fragile.^[^ ^26^ ^]^ This property suggests that our material may be suitable in industrial‐level applications that require fluidized beds. CO_2_ activation time and temperature were also investigated (Figure S11–S14, Supporting Information). It turns out that 9‐h activation at 850 °C is the optimized condition for balanced surface area and bulk density of carbon flowers.

### 2.2 Formation Mechanism

The hypothesized formation process of the freestanding PANF pellets is shown in Figure 2a based on observations shown in Figure 2 and Figure S6 and S7, Supporting Information. The polymerization reaction started out as a homogeneous solution, in which polyacrylonitrile began forming and precipitated once the polymer chain reached a critical length governed by its solubility. Furthermore, these precipitates may serve as nuclei to enable the formation of larger structures. As the reaction proceeded, more polymer precipitates were produced and aggregated on the nuclei, constituting the building blocks for PANF. With the increasing amount of building blocks formed, they gradually self‐assembled into PANF structures, and over time the particles became denser and slightly increased in size (Figure S7, Supporting Information). As the narrowly dispersed PANF particle dispersion got more concentrated, the PANF particles began to self‐assemble into more densely packed arrays and eventually formed a freestanding pellet after solvent was absorbed and evaporated.

We also observed that the PANF particles share similarities with concentrated colloidal spheres. For example, their particle sizes are all in the submicrometer range, their size distribution is narrow, and the typical concentrations of particles are high. The final structure of the PANF arrays also resembles common colloidal crystals. However, it is a surprising finding that the flower‐like spheres still assemble into a similar structure as regular spheres. First of all, the particle shapes could significantly influence the self‐assembly process, which may form totally different packing structures compared with regular close‐packed sphere arrays. Even a minor change of the particle's spherical geometry could result in a different final structure. For example, a lock‐and‐key structure could be obtained by designing matching areas on different particles.^[^ ^27^ ^]^ A more similar example to our PANF particles was the octapod‐shaped nanoparticles.^[^ ^28^ ^]^ The branched nanopods introduce extra short‐ranged pod–pod interactions in addition to the longer‐ranged interparticle interactions, leading to the formation of interlocked linear chains. In addition, the complex shape of the particles could restrict the translational and rotational degree of freedom in neighboring nanoparticles, which may trap the crystallization process in “jammed” amorphous structures. However, PANF particles show no interlocking behavior or trapped assembly structure throughout the formation process, despite that the branched structure forms at very early stage of the reaction (Figure S7, Supporting Information). We find that as long as the PANF particles have low polydispersity and roughly spherical shapes, the final structure turns to be similar to colloidal crystals formed by spheres. The results indicate that branched particles could still have similar colloidal behavior to the regular hard spheres. Second, the self‐assembly process of PANF is much more complicated than conventional colloidal self‐assembly, in which particle formation and self‐assembly are two individual processes. However, in our systems, the formation of individual PANF particles and the PANF arrays are carried out at the same time. It is difficult to predict the final structure of such a complicated system. Interestingly, such complicated systems could exhibit simple assembly behavior like smooth spheres. Therefore, we attempt to rationalize the formation of PANF arrays following the phase transition behavior previously described for concentrated hard colloidal spheres.^[^ ^29^ ^]^ It was previously reported that when the suspensions of monodispersed hard spheres reached a certain volume fraction, they tend to crystallize, forming face‐centered cubic arrays.^[^ ^29^ ^]^ Dilute hard sphere suspensions are unable to crystallize due to the large interparticle distance, where the particles could freely move around their neighbor by Brownian motion, showing liquid‐like feature. On the contrary, if the concentration is too high, the ordered structures would not be formed either. In this case, even though the crystalline state is thermodynamically preferred, the interparticle distance is too close such that the particle movement is hindered from forming colloidal crystals within a limited time scale. To validate this hypothesis, reactions at different monomer concentrations were subsequently conducted. Figure 2b shows the SEM image of CF arrays made from different monomer concentrations (36, 44, 50, 57, and 66 vol%, respectively). Indeed, ordered arrays could not be obtained when the concentration was either too low or too high, which is consistent to the observations for the colloidal hard‐sphere systems.^[^ ^29^ ^]^ In addition to acetone, the ordered arrays could also be obtained from a variety of other solvents in the same concentration range. For example, our previous reports showed that polyacrylonitrile–tetrahydrofuran (THF) and polyacrylonitrile–pyridine have similar interactions to polyacrylonitrile–acetone.^[^ ^18^ ^]^ These solvents should be able to also produce carbon flower particles with similar morphology. Our experiments confirmed our assumption that all three solvents in THF, pyridine, and acetone could produce ordered PANF arrays at a similar concentration range, as shown in our obtained SEM images of CFP synthesized from acetone, THF, and pyridine, respectively (Figure 2c–e and Figure S8, Supporting Information).

### 2.3 Improvement of Volumetric Surface Area

**Figure 3:**
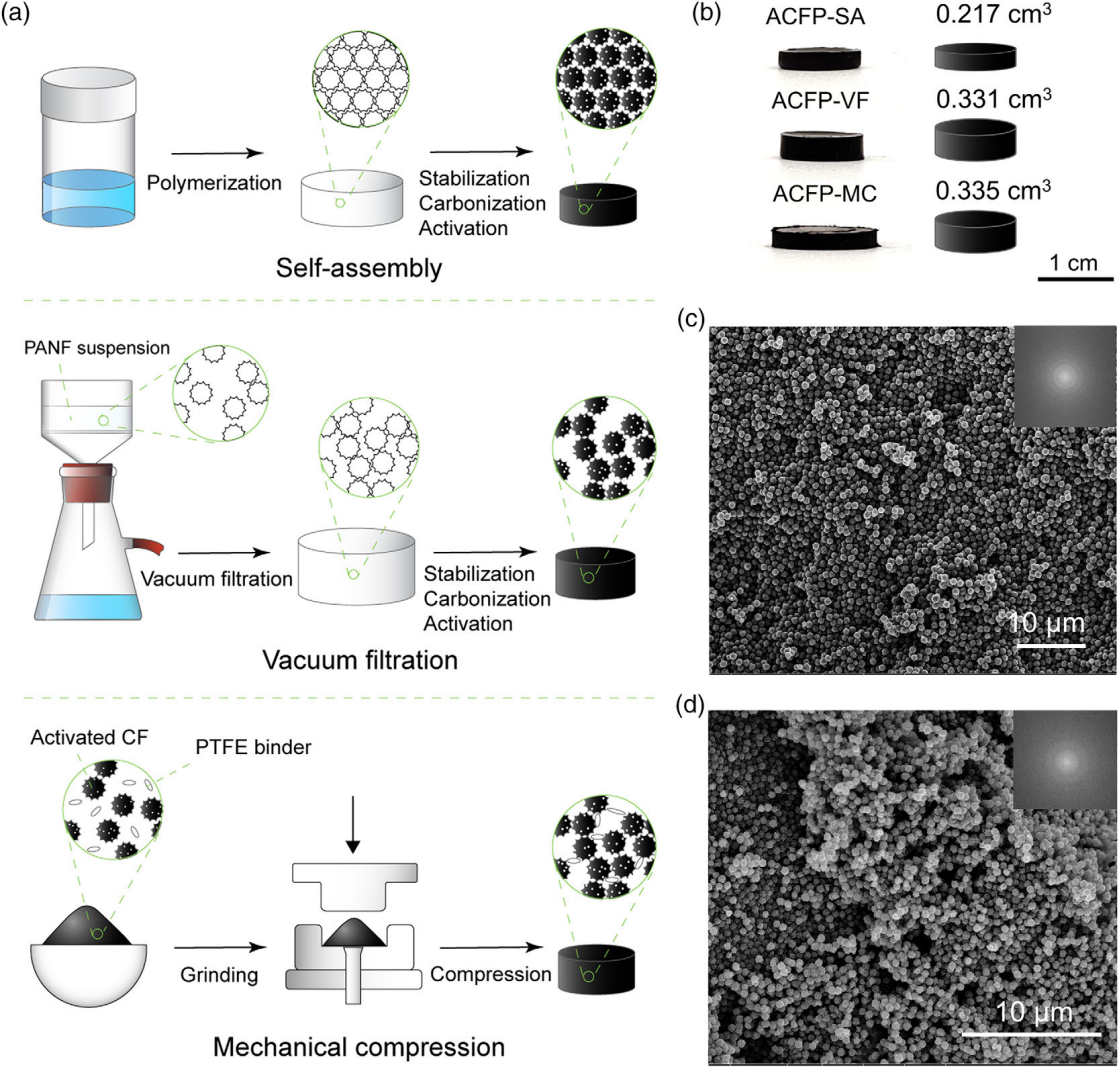
a) Schematic diagrams showing the ACFP synthetic process by self‐assembly, vacuum filtration, and mechanical compression. b) Volume difference of ACFP‐SA, ACFP‐VF, and ACFP‐MC with the same weight. A photo of ACFPs with the same weight (left). Values show the volume calculated based on the photo. Cylinders with the same diameter and different heights based the corresponding volume (right). c) An SEM image of an ACFP‐VF sample with an FFT image on the top‐right corner. d) An SEM image of an ACFP‐MC sample with an FFT image on the top‐right corner.

Due to the ordered structure of carbon flower arrays, we reasoned that interparticle free volume caused by inefficient packing could be minimized to significantly reduce the volume of the bulk material. Therefore, the bulk density and volumetric performance of the material could both then be improved. To determine the degree of improvement of volumetric performance by dense packing, we synthesized ACFP using three different approaches: self‐assembly, vacuum filtration, and mechanical compression (**Figure** **3**a). ACFPs from self‐assembly (ACFP‐SA) were synthesized using the procedures presented in this work. Next, ACFP from vacuum filtration (ACFP‐VF) were synthesized by vacuum filtrating PANF suspensions and the following heat treatment. Finally, ACFP from mechanical compression (ACFP‐MC) were synthesized from mechanical compressing (20 MPa) of activated carbon flower powders and polytetrafluoroethylene (PTFE) binder. The morphologies of ACFP‐VF and ACFP‐MC are shown in Figure 3c,d and Figure S9, Supporting Information. We observed that the particles are randomly packed in the bulk materials made from the last two methods, with plenty of voids in between particles. We further confirmed this observation, as shown in Figure 3b, which depicts the volume difference, as evidenced from the height difference, of ACFP‐SA, ACFP‐VF, and ACFP‐MC with the same mass and same diameter. As a result of substantially large interparticle‐free volume caused by random packing, both ACFP‐VF and ACFP‐MC have a larger volume compared with ACFP‐SA, again showing that proper assembly method is important to obtain ordered packing to a minimized interparticle‐free volume.

**Figure 4:**
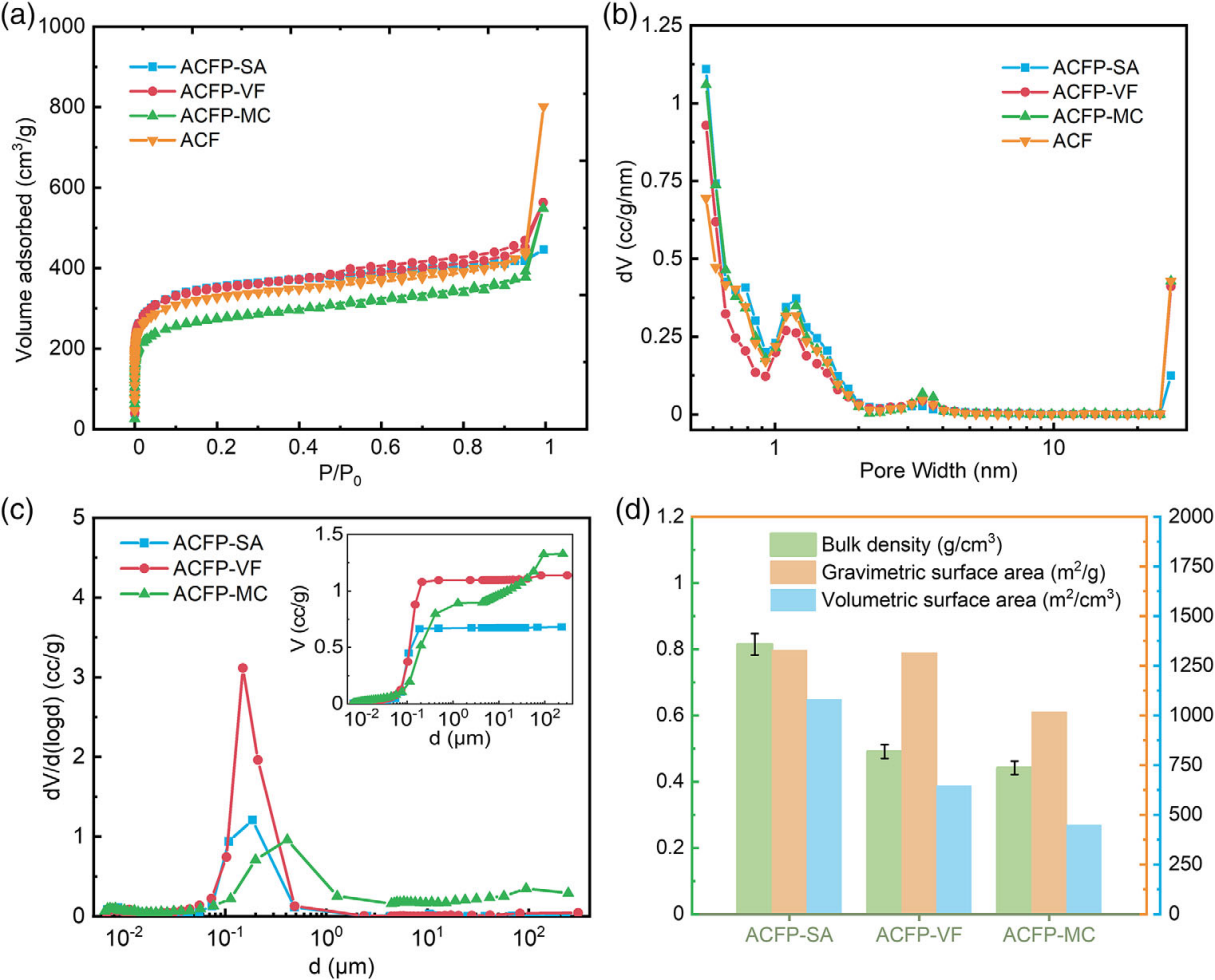
a) N_2_ adsorption/desorption isotherms of ACFP‐SA, ACFP‐VF, ACFP‐MC, and ACF. b) Pore size distribution calculated from QSDFT theory with carbon model of slit pores. c) The pore size distribution of ACFP‐SA, ACFP‐VF, and ACFP‐MC obtained from mercury porosimeter. The inset shows the cumulative pore volume of the three samples obtained from mercury porosimeter. d) Differences in bulk density, gravimetric surface area, and volumetric surface area between ACFP‐SA, ACFP‐VF, and ACFP‐MC.

The pore structures were further characterized by N_2_ adsorption/desorption experiments and mercury porosimeter. N_2_ adsorption/desorption is widely used in characterizing Brunauer–Emmett–Teller (BET) surface area, the pore size distribution of micropore, mesopore, and small macropores. However, due to limitations in the principle and instrumentation, pores larger than ≈380 nm, as determined by the Kelvin equation,^[^ ^30^ ^]^ could not be detected but may constitute the majority of interparticle pores. Instead, mercury porosimeter is usually used to characterize large mesopores and macropores, as it could measure pores with diameters up to 1 mm. Therefore, we proceeded to analyze the pore structure of ACFPs using both methods. **Figure** **4**a shows the N_2_ isotherm curves of different samples. The obtained isotherms displayed a combined shape of type I and type II isotherms, indicating the presence of micropores and mesopores produced by CO_2_ activation and macropores from our carbon flower structures.^[^ ^30^ ^]^ The rapid ramp‐up at the point near 0.995 standard pressure is caused by condensation of liquid nitrogen in macropores. The larger the ramp‐up, the larger the macropore volumes. We observed that ACFP‐SA has almost the same isotherm as ACFP‐VF and its powder analog activated carbon flowers (ACFs) with the exception for the last point, indicating both materials have similar micropore and mesopore distribution and BET surface area. The difference at the last point suggests that ACFP‐SA has fewer macropores as a result of efficient packing. The result also suggested that mechanical compression may have resulted in damages to the particles because there is an apparent drop in the N_2_ adsorption amount of ACFP‐MC, as compared with the other three materials. Figure 4b shows the pore size distributions as measured by N_2_ adsorption. The obtained plots showed that the ACFP‐SA, ACFP‐VF, ACFP‐MC, and ACF all have very similar micropore and mesopore size distributions, indicating dense packing did not result in any damages to the pores. In addition to micropores and mesopores, pores with submicrometer diameters were observed to also be prevalent, as shown in Figure 4c. As ACFP‐SA does not have many pores >1 μm diameter, and considering our particle size is ≈1 μm, we concluded that their interparticle distance is small. The submicrometer pore sizes resulted from its particle structures and small interparticle distances. However, ACFP‐VF has a much larger pore volume in the submicrometer pore region compared with ACFP‐SA, indicating the existence of a large interparticle‐free volume. In addition, pore size distribution of ACFP‐MC is much broader macropore distribution compared with ACFP‐SA and ACFP‐VF. It has plenty of pores >1 μm diameter, as shown in Figure 4c. As a result, the total pore volume of ACFP‐SA is significantly smaller compared with ACFP‐VF and ACFP‐MC, as shown in the inset of Figure 4c.

Due to the different pore structures in the macropore region, the bulk densities of ACFPs are expected to be different. Figure 4d shows bulk density, gravimetric surface area, and volumetric surface area of ACFPs. Measurement of bulk density was determined based on a modified Archimedes’ principle, which is further discussed in the Experimental Section. We believe this is one of the most accurate methods to determine bulk density of such materials without large overestimation as discussed later. The gravimetric surface area relied on the obtained value of the BET surface area. Due to the microporous feature of ACFPs, calculation of the BET surface area based on the usual 0.05–0.35 *P*/*P*_0_ range is inaccurate. Point selection for calculating the BET surface was adjusted according to Rouquerol method,^[^ ^31^ ^]^ as shown in Figure S10, Supporting Information. The volumetric surface area is the product of bulk density and the gravimetric surface area. The gravimetric surface area values of ACFP‐SA and ACFP‐VF are 1326 and 1315 m^2^ g^−1^, respectively. These results indicated that self‐assembly did not damage the particle structures and thus the gravimetric performance was maintained. However, ACFP‐MC has measured to have only a 1017 m^2^ g^−1^ gravimetric surface area, whereas the ACF powder has a 1226 m^2^ g^−1^ gravimetric surface area, indicating that its structures have collapsed, along with blocked pores, potentially caused by mechanical compression and PTFE binders. Due to the significantly reduced interparticle‐free volume in ACFP‐SA, it now has a much higher bulk density (0.815 g cm^−3^) as compared with ACFP‐VF (0.491 g cm^−3^) and ACFP‐MC (0.442 g cm^−3^). Therefore, the volumetric surface area of ACFP‐SA (1081 m^2^ cm^−3^) is 65% higher compared with ACFP‐VF (656 m^2^ cm^−3^) and 140% higher compared with ACFP‐MC (450 m^2^ g^−1^).

In summary, our obtained results indicate that our strategy in synthesizing self‐assembling and highly ordered carbon particles will reduce the interparticle‐free volume and increase the volumetric performance of the material, without affecting its gravimetric performance. We also note that vacuum filtration of PANF is able to form freestanding pellets without the need for additional binders. Even though only randomly packed particles were obtained at this time, we believe that ordered structures may be obtained upon further optimization in filtering conditions.

### 2.4 Densely Packed Carbon Sphere Pellets

**Figure 5:**
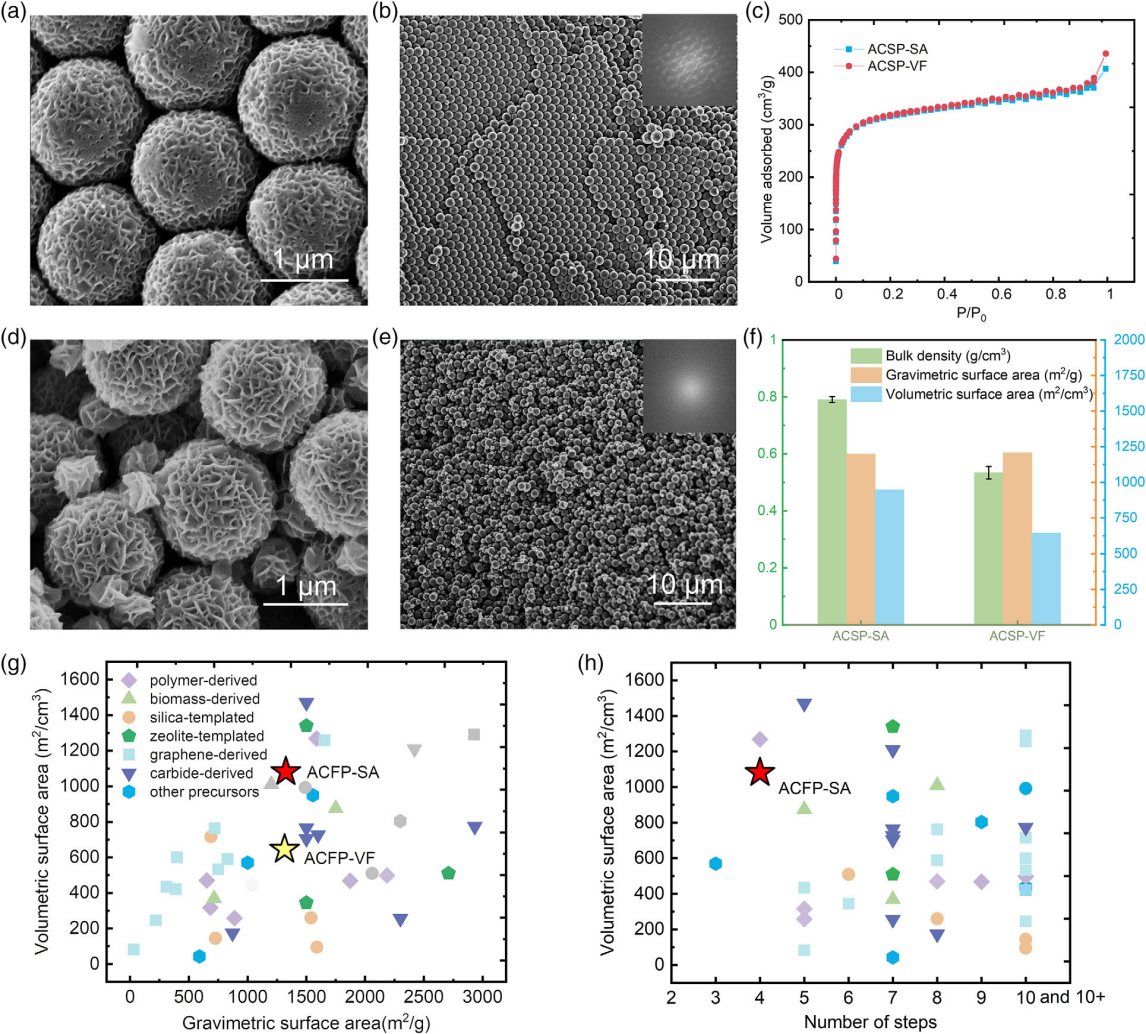
a,b) SEM images of CSs pellet by self‐assembly (CSP‐SA) with an FFT image on the top‐right corner of (b). c) N_2_ adsorption/desorption isotherms of ACSP‐SA and ACSP‐VF. d,e) SEM images of CSP‐SA with an FFT image on the top‐right corner of (e). f) Differences in bulk density, gravimetric surface area, and volumetric surface area between ACSP‐SA and ACSP‐VF. g) Comparison of surface areas with carbon materials reported in the literature.^[^ ^7^, ^10^, ^11^, ^13^, ^14^, ^15^, ^17^, ^32^, ^33^, ^34^, ^35^, ^36^, ^37^, ^38^, ^39^, ^40^, ^41^, ^42^, ^43^, ^44^, ^45^, ^46^, ^47^, ^48^, ^49^, ^50^, ^51^, ^52^, ^53^, ^54^, ^55^, ^56^, ^57^, ^58^, ^59^, ^60^, ^61^, ^62^, ^63^, ^64^ ^]^ Data points with a light gray color are calculated based on the density of graphite and total pore volume measured by N_2_ adsorption/desorption experiment, which may have overestimated the actual density and was not reported. Detailed references and values are shown in Table S1, Supporting Information. h) Comparison of volumetric surface areas and the number of synthesis steps with carbon materials reported in the literature.^[^ ^7^, ^10^, ^11^, ^13^, ^14^, ^15^, ^17^, ^32^, ^33^, ^34^, ^35^, ^36^, ^37^, ^38^, ^39^, ^40^, ^41^, ^42^, ^43^, ^44^, ^45^, ^46^, ^47^, ^48^, ^49^, ^50^, ^51^, ^52^, ^53^, ^54^, ^55^, ^56^, ^57^, ^58^, ^59^, ^60^, ^61^, ^62^, ^63^, ^64^ ^]^ Some data points have been added three extra steps accounting for the synthesis of commercial carbon precursors (Table S1, Supporting Information).^[^ ^11^, ^13^, ^15^, ^32^, ^33^, ^34^, ^37^, ^39^, ^47^, ^49^, ^52^, ^53^, ^54^, ^55^, ^57^, ^58^, ^59^, ^60^, ^61^, ^63^, ^64^ ^]^

In our previous report, we had observed that adding a small amount of styrene during the polymerization step enabled the morphology of the PANF particles to be spherical.^[^ ^18^ ^]^ Here, we explore the packing effects of these spherical particles to demonstrate the synthesis‐assembly method also applies to other monomers. To synthesize carbon spheres (CSs), 2.5% vol of styrene was added in the solution of polymerization, with all other conditions remaining the same. We observed the CS could be converted to a freestanding carbon sphere pellet (CSP), and the obtained morphology is shown in **Figure** **5**a,b. We also observed that CS could further self‐assemble into ordered arrays with reduced interparticle‐free volume. Vacuum‐filtrated pellets could also be fabricated using polyacrylonitrile sphere (PANS) suspension, which is composed of randomly packed particles (Figure 5d,e). Next, Figure 5f shows the bulk density and surface area differences of activated CSs synthesized through the two techniques of self‐assembly (ACSP‐SA) and vacuum filtration (ACSP‐VF). Our obtained results suggest that the bulk density of CSP can be improved through ordered particles packing, without affecting the gravimetric surface area.

### 2.5 Performance and Discussion

A comparison of the surface area of our prepared samples with different carbon materials is shown in Figure 5g and Table S1, Supporting Information, including graphene‐derived carbon, biomass‐derived carbon, polymer‐derived carbon, silica‐templated carbon, and zeolite‐templated carbon.^[^ ^7^, ^10^, ^11^, ^13^, ^14^, ^15^, ^17^, ^32^, ^33^, ^34^, ^35^, ^36^, ^37^, ^38^, ^39^, ^40^, ^41^, ^42^, ^43^, ^44^, ^45^, ^46^, ^47^, ^48^, ^49^, ^50^, ^51^, ^52^, ^53^, ^54^, ^55^, ^56^, ^57^, ^58^, ^59^, ^60^, ^61^, ^62^, ^63^, ^64^ ^]^ For ACFP‐VF, both gravimetric and volumetric performances are not outstanding among many of the materials that have developed. However, due to self‐assembly, the volume of ACFP‐SA is rendered much smaller and thus has a much higher volumetric surface area (1081 m^2^ cm^−3^). We want to point out that the data points with a light gray color in Figure 5g are based on bulk densities calculated from total pore volume measured by N_2_ adsorption/desorption based on Equation (1).^[^ ^32^, ^33^, ^34^, ^35^, ^36^, ^53^, ^54^, ^59^ ^]^ Their actual bulk densities were not reported, which are usually smaller than the calculated bulk densities based on only N_2_ adsorption/desorption data as the larger pores are not measured by this method.

(1) $$\rho_{\text{bulk}}=\frac{1}{V_{\text{pore}}+\frac{1}{\rho_{\text{carbon}}}}$$

The total pore volume ($V_{\text{pore}})$ obtained from N_2_ adsorption/desorption can only measure the total pore volume from pores smaller than ≈380 nm (depending on the pressure used for calculating total pore volume). In most randomly packed particle systems, the interparticle pores are usually >380 nm, resulting in underestimation of the actual total pore volume using this approach. In addition, the true density of carbon does not include the existence of closed pores in the material, which may again result in an underestimation of the material volume. Through the aforementioned rationale, volumetric surface areas may again have been overestimated.

It is also important to simplify the synthetic process steps for reliability and cost issues. As shown in Figure 5h and Table S1, Supporting Information, even though the volumetric surfaces of some of these reported porous carbon materials are higher than ACFP‐SAs,^[^ ^15^, ^64^ ^]^ their synthetic processes may involve multiple steps which will increase the complexity and cost for future large‐scale production. In addition, many high surface‐area porous carbon materials use other commercially available products, e.g., graphene oxide (GO) and commercial porous carbon,^[^ ^37^ ^]^ which may involve a few synthetic steps to obtain these more costly starting materials. To our best knowledge, we added three extra steps (synthesis, carbonization, and mixing and separation) for these materials.^[^ ^11^, ^13^, ^15^, ^32^, ^33^, ^34^, ^37^, ^39^, ^47^, ^49^, ^52^, ^53^, ^54^, ^55^, ^57^, ^58^, ^59^, ^60^, ^61^, ^63^, ^64^ ^]^ However, achieving the final materials may be more complex than what is shown in Figure 5h. In this described work, our synthesis of ACFP‐SA comprised a one‐pot polymerization step from readily available commercial monomer and subsequent well‐established thermal treatment procedures. For large‐scale industrial production, the stabilization, carbonization, and activation steps can be carried out in a single process flow, achieved by simply setting a combined temperature profile and automatic gas (air, N_2_, and CO_2_) switching at certain temperatures.

Importantly, while the volumetric surface area is a good standard to compare the potential volumetric performance among different materials, it is however not the only parameter to determine its final volumetric performance. There are several other parameters that may also influence the overall performance, such as the physical structure of pores, connectivity of the pores, heteroatom doping, conductivity, and the practical application where the material is used. A minor difference in the volumetric surface does not necessarily suggest the overall performance will be different. In an ideal case, the highest volumetric surface area can be attained when there are only narrowly dispersed micropores in the material. Nevertheless, many advantageous features will be compromised, such as pore connectivity, hierarchical structure, and pore tortuosity. Therefore, it is essential to balance the volumetric performance with their original properties of the material. We observed that self‐assembly of carbon flower is able to greatly improve the volumetric surface area without affecting its original micro‐ and mesopore structures. The hierarchical porous structure is still well preserved, which is beneficial for applications requiring fast diffusion of molecules inside the material. In addition, our developed CFPs represent a simple synthetic process, tunable monolith shapes, high mechanical stability, tunable heteroatom doing, and versatile structures,^[^ ^18^, ^21^ ^]^ which make them stand out among high‐bulk‐density carbon materials. Therefore, they are promising candidates in many applications such as batteries, gas storage, and supercapacitors.

**Figure 6:**
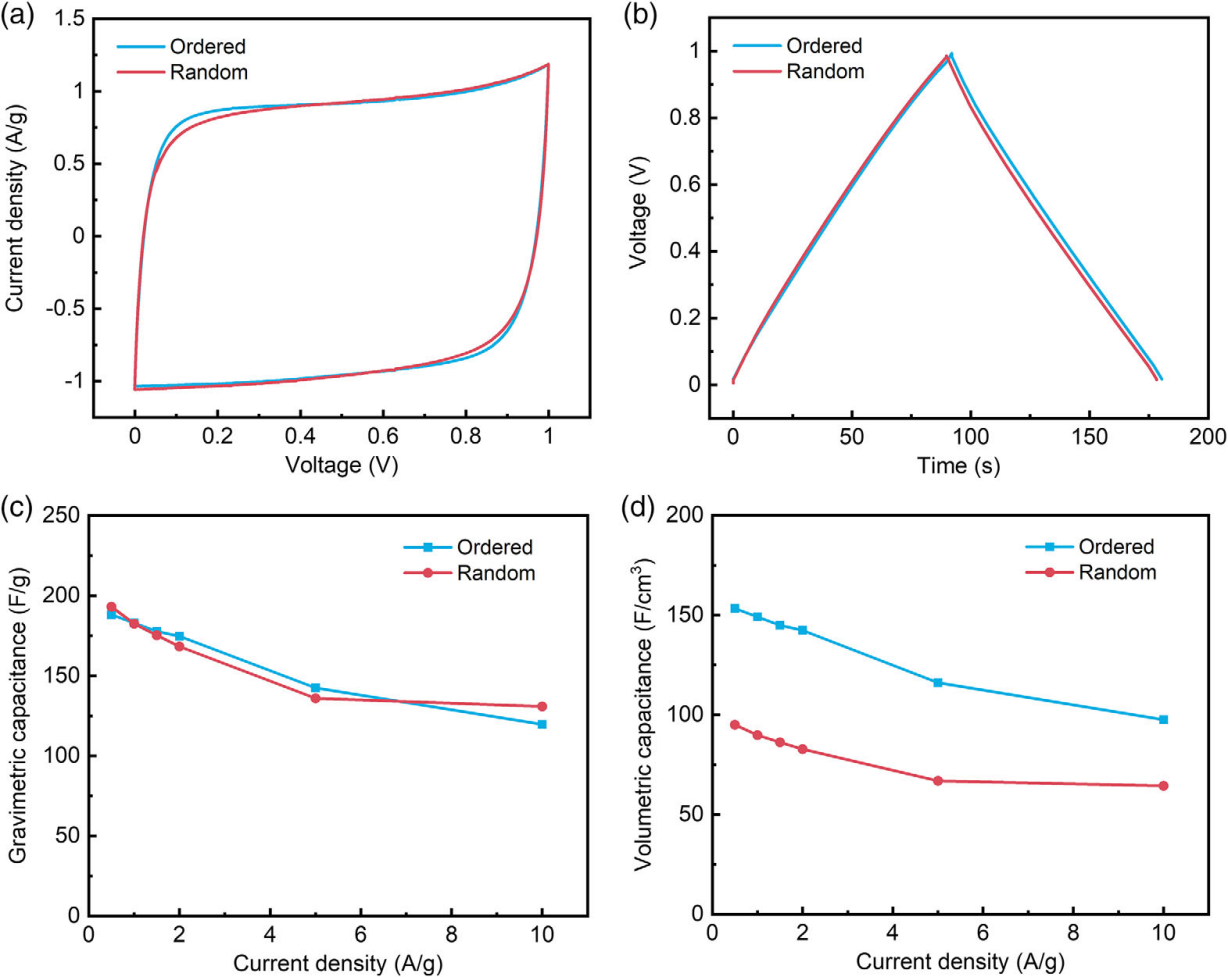
a) CV curves at a scan rate of 10 mV s^−1^. b) GCD curves at a current density of 1 A g^−1^. c) Gravimetric capacitances versus current densities. d) Volumetric capacitances versus current densities.

Previously, we have shown that this densely packed and highly ordered structures have advantages for methane storage.^[^ ^19^ ^]^ Herein, we use supercapacitor as an example to demonstrate the advantages of orderly packed versus randomly packed carbon flower particles. Freestanding ACFP‐SA (ordered) and ACFP‐VF (random) samples were cut into thin sheets for electrochemical measurement (Figure S17, Supporting Information). Supercapacitors were assembled in a symmetric two‐electrode configuration using two equal‐weight sheets of the same material. **Figure** **6**a,b shows the cyclic voltammetry (CV) and galvanostatic charge–discharge (GCD) curves of the supercapacitors. The quasi‐rectangular CV curves and isosceles triangular GCD curves indicate nearly ideal capacitive behavior. Carbon flowers with ordered packing and random packing have the nearly coincident CV and GCD curves under the experiment conditions, indicating similar gravimetric supercapacitor performances. Figure 6c shows gravimetric capacitance of ordered and random packing derived from GCD curves (Figure S18, Supporting Information) at different current densities. It is evident that orderly packed and random‐packed carbon flowers have similar gravimetric capacitance due to their nearly identical gravimetric surface areas. However, as the density of ACFP‐SA is much higher than ACFP‐VF, ACFP‐SA shows a clear advantage in volumetric capacitance (up to 153 F cm^−3^), as shown in Figure 6d. The obtained results reinforce that orderly packing porous particles could significantly reduce the bulk volume of the materials and improve the volumetric performance.

## 3 Conclusion

In summary, we have developed a simple approach to self‐assemble PANF particles in centimeter‐scale ordered arrays, forming freestanding and binder‐free pellets. Our synthesized carbon pellets have good mechanical strength, tunable shapes, and are able to preserve the original morphology of individual particles. Importantly, our densely packed particles are observed to have much smaller interparticle free volume than randomly packed particles, which resulted in significantly reduced material volume and increased bulk density. The volumetric surface area of the materials showed a 65–140% improvement as compared with randomly packed particles derived from vacuum filtration and mechanical compression, while suffering no decrease in gravimetric surface area. An ultrahigh volumetric surface area (1081 m^2^ cm^−3^) is thus achieved. In addition, the orderly packed carbon flowers exhibit significant improvement in volumetric capacitance (up to 153 F cm^−3^) compared to the randomly packed carbon flowers (up to 95 F cm^−3^). Our obtained result underscores the importance in controlling particle packing within porous materials to reduce the bulk volume of the material. Upon optimization, this versatile strategy should be applicable to improve the volumetric performance of similar material systems. Moreover, the densely packed carbon flower materials possess many advantageous properties, including hierarchical pore structure, simple synthetic procedure, tunable heteroatom doping, high mechanical stability, and versatile structures,^[^ ^18^, ^21^ ^]^ making them promising candidates for environment, energy, and electronic applications.

## 4 Experimental Section

4.1

4.1.1

##### Synthesis of Freestanding Pellets with Densely Packed Carbon Flowers (ACFP‐SA)

PANFPs were prepared by a free radical polymerization with acrylonitrile as the monomer, acetone as the solvent, and AIBN as the initiator. Typically, 0.5 mL acrylonitrile, 0.5 mL acetone, and 0.5 mg AIBN were added into an air‐tight vial and were heated at 70 °C with N_2_ protection for 3 h to overnight. The one‐pot reaction produced PANF particles, and they could self‐assemble into ordered arrays, forming freestanding PANFPs during the reaction. PANFPs were then dried under vacuum at 50 °C overnight to remove the remaining monomer and solvent. To convert PANFPs to CFPs, they were first stabilized in the air at 230 °C for 4 h with a ramping rate of 0.1 °C min^−1^. The stabilized PANFPs were then carbonized to CFPs at 600 °C for 2 h in nitrogen (70 sccm) with a ramping rate of 2 °C min^−1^. CO_2_ activation was applied to CFPs to further increase their surface areas. In particular, CFPs were heated up to 850 °C with a ramping rate of 5 °C min^−1^ and held for 9 h in CO_2_ environment (50 sccm).

##### Synthesis of Freestanding Pellets by Vacuum Filtration (ACFP‐VF)

PANF suspensions were prepared by a free radical polymerization with acrylonitrile as the monomer, acetone as the solvent, and AIBN as the initiator. Typically, 2 mL acrylonitrile, 10 mL acetone, and 2 mg AIBN were added into an air‐tight vial and were heated at 70 °C with N_2_ protection for overnight. The as‐prepared suspensions were vacuum filtrated by a vacuum filter assembly with a filter paper of 0.4 μm pores. Freestanding pellets formed after vacuum filtration. Then the pellets were dried under vacuum at 50 °C for overnight to remove the remaining monomer and solvent. The subsequent stabilization, carbonization, and activation steps followed the same procedure as the case for PANFPs.

##### Synthesis of Freestanding Pellets by Mechanical Compression (ACFP‐MC)

PANF powder was prepared by a free radical polymerization with acrylonitrile as the monomer, acetone as the solvent, and AIBN as the initiator. Typically, 5 mL acrylonitrile, 5 mL acetone, and 5 mg AIBN were added into an air‐tight vial and were heated at 70 °C with N_2_ protection for 2 h. The as‐prepared powders were vacuum dried at 50 °C overnight to remove the remaining monomer and solvent. The subsequent stabilization, carbonization, and activation steps followed the same procedure as PANFPs. After activation, the powder was mixed with 10 wt% of PTFE and ground by a mortar and pestle. Then it was compressed by an MTI Hydraulic Pellet Press at 20 MPa for 2 min.

##### Synthesis of CS Pellets (ACSP‐SA and ACSP‐VF)

ACSP‐SAs were prepared in the same procedure as ACFP‐SAs, except a small amount of styrene was mixed with the monomer. Typically, 0.4875 mL acrylonitrile, 0.0125 mL styrene, 0.5 mL acetone, and 0.5 mg AIBN were added into an air‐tight vial and were heated at 70 °C with N_2_ protection for 3 h to overnight. The following heat treatment is the same as ACFP‐SAs’. PANS suspensions were prepared by free radical polymerization of 4.875 mL acrylonitrile, 0.125 mL styrene, 10 mL acetone, and 5 mg AIBN. ACSP‐VFs were prepared using CS suspensions following the same procedure for producing ACFP‐VFs.

##### Characterization

Bulk density ($\rho_{\text{bulk}}$) was simply a quotient of mass and bulk volume by definition. While the measurement of mass was simple, measurement of volume was not straightforward when the geometry of the material was not standard. As the shape of ACFPs was not a standard cylinder, it was not easy to measure the geometric volume of the materials without producing non‐negligible errors. Meanwhile, it was also invalid to simply apply the Archimedes’ principle to measure the materials’ volume because liquid like water cannot wet ACFPs, and they could either form bubbles on the materials’ surface or partially occupied the pores. Therefore, we measured the bulk density of ACFPs by a modified Archimedes’ principle. Specifically, ACFPs were first vacuum dried overnight and then put in isopropanol (IPA) for 1 h to fully absorb IPA. As IPA can wet ACFP, it could go inside the pores and could occupy the volume of the pores. By measuring the change of IPA volume using the Archimedes’ principle, we could get the skeletal volume of the material ($V_{\text{skeletal}}$), which was mainly the volume of the carbon. After that, the mass change of material before and after IPA absorption (Δm) was recorded, from which we could calculate the volume of IPA being absorbed. The bulk density of ACFPs could be obtained as follows

(2) $$\rho_{\text{bulk}}=\frac{m}{V_{\text{skeletal}}+\frac{\Delta m}{\rho_{\text{IPA}}}}$$

SEM images were collected by an FEI Magellan 400 XHR SEM at Stanford Nano Shared Facilities. Mercury porosimeter measurement was carried out at a Quantachrome Poremaster 33 at Stanford Nano Shared Facilities. N_2_ adsorption was conducted on Quantachrome Autosorb iQ3 with 99.999% N_2_ at 77 K at Stanford Nano Shared Facilities. All samples were degassed overnight before measurement. Pore size distributions were obtained using quenched solid‐state density functional (QSDFT) calculations with the carbon model of slit pores. The BET surface area was calculated within the pressure range of *P*/*P*_0_ = 0.008–0.1 (Figure S10, Supporting Information).

##### Supercapacitor Fabrication and Characterization

Freestanding ACFP‐SA and ACFP‐VF were cut into thin sheets and pressed onto glassy carbon electrodes without binders. Supercapacitors were assembled in a symmetric two‐electrode configuration using two equal‐weight sheets of the same material. Two working electrodes with a separator in between were assembled into a sandwich‐like structure with 1 m H_2_SO_4_ as electrolyte. All the electrochemical measurements were carried out on a Biologic MPG‐2 workstation at room temperature (25 °C). The gravimetric capacitance *C*_wt_ was derived from GCD curves through the following equation

(3) $$C_{\text{wt}}=\frac{2\left(I\times \Delta t\right)}{m\times \Delta U}$$

where *I* is the discharging current, Δt is the discharging time, *m* is the mass of the material on the electrode, and ΔU is the voltage change after excluding the infrared drop during the discharging process. The corresponding volumetric capacitance *C*_vol_ was calculated through

(4) $$C_{\text{vol}}=C_{\text{wt}}\times \rho_{\text{bulk}}$$

## Conflict of Interest

The authors declare no conflict of interest.

## Data Availability Statement

The data that support the findings of this study are available from the corresponding author upon reasonable request.

## Supporting information

Supplementary Material

Supplementary Material

Supplementary Material
