# Supplementary material for: Densely Packed and Highly Ordered Carbon Flower Particles for High Volumetric Performance
Source: Small Sci. 2021 Mar 14;1(7):2000067. doi: 10.1002/smsc.202000067 (PMC11935817; doi:10.1002/smsc.202000067)
Supplement: Supplementary file 1 — Supplementary Material [file SMSC-1-2000067-s001.pdf]

Supporting Information

Huaxin Gong, Shucheng Chen, Rui Ning, Ting-Hsiang Chang, Jeffrey B.-H. Tok, Zhenan Bao[*]

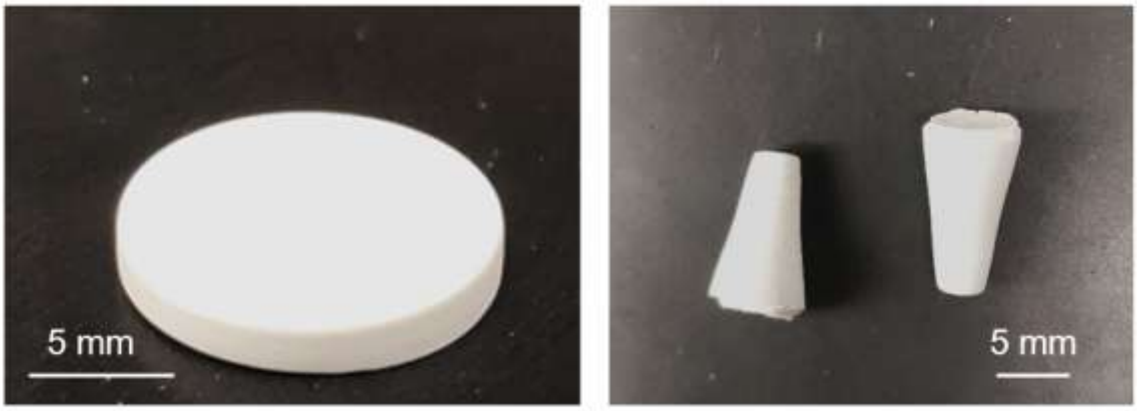

Figure S1. Tunable shapes of PANFP

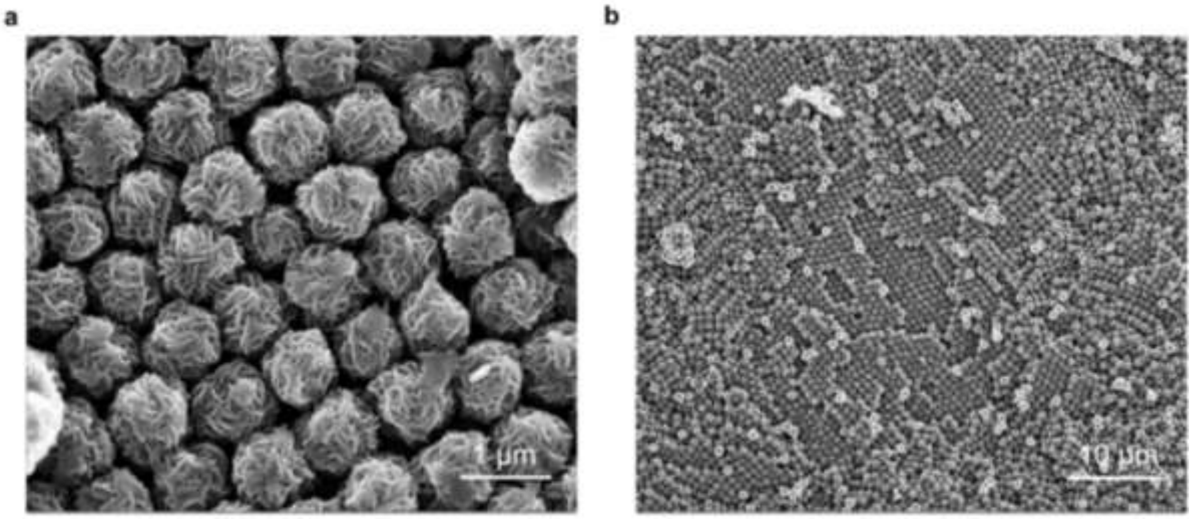

Figure S2. SEM images of a CFP before CO_2 activation

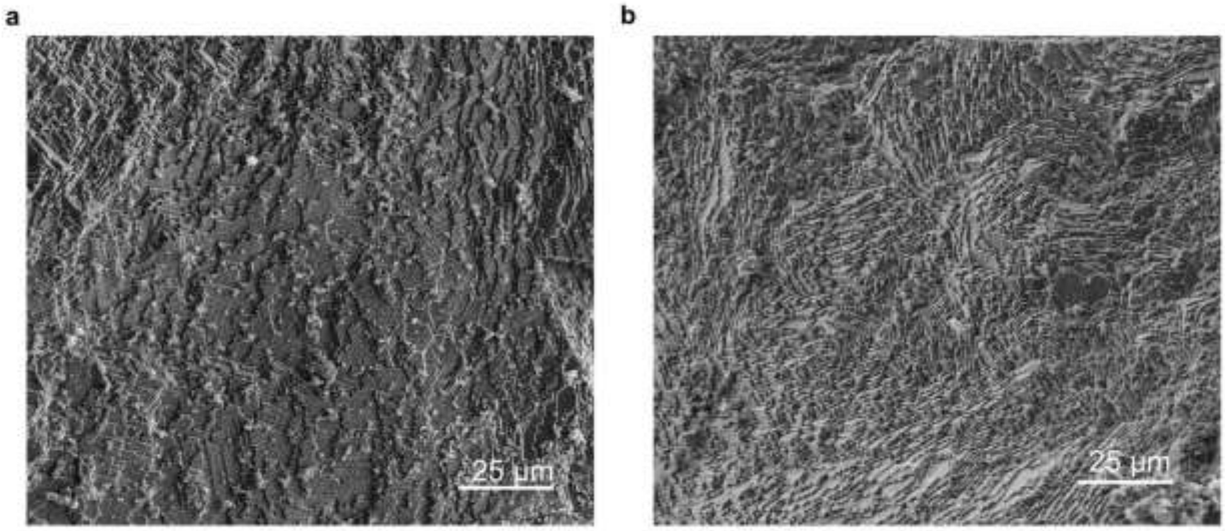

Figure S3. Low-magnification SEM images of an ACFP-SA taken from two different angles, showing a wider view of the ordered structure

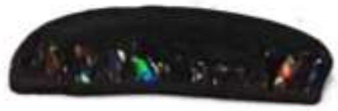

Figure S4. Photo of the cross-section of an ACFP-SA sample, showing the ordered structures exist throughout the sample

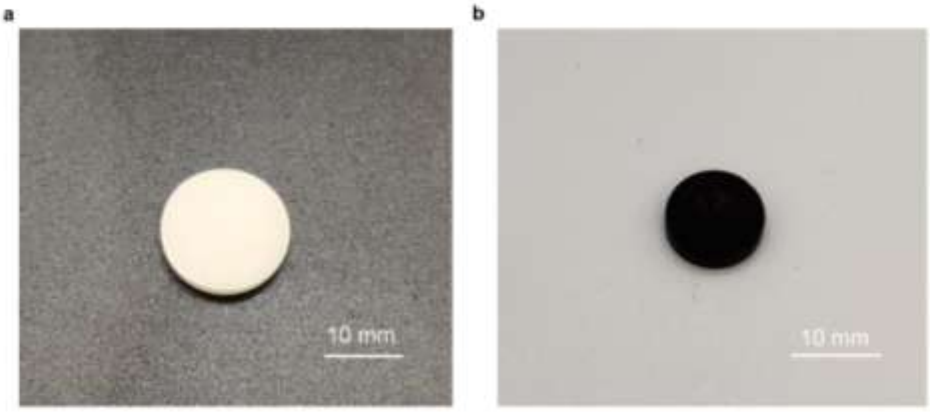

Figure S5. (a) A photo of a PANFP sample after 10 N (~127 kPa) compression (b) A photo of an ACFP-SA sample after 10 N (~127 kPa) compression

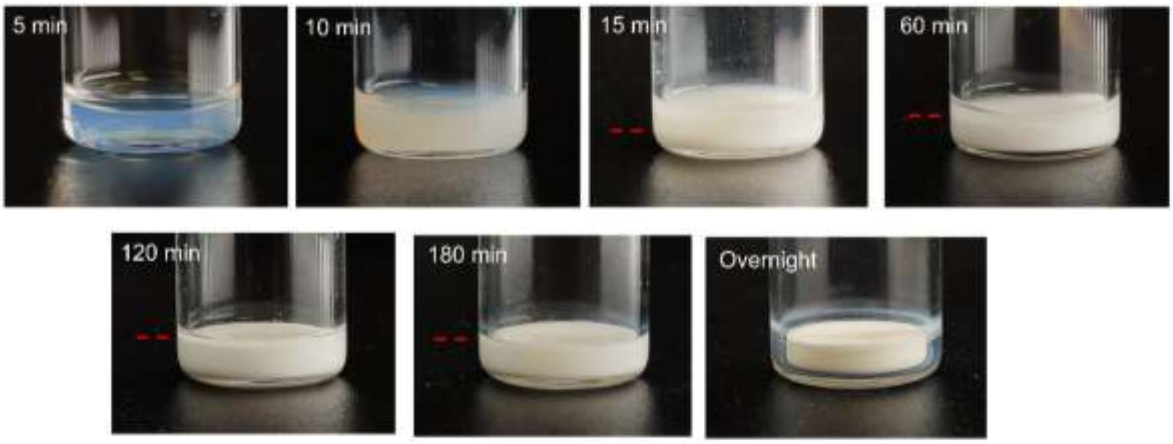

Figure S6. Photos showing different stages of formation process of a PANFP after starting the reaction by heating to 70°C. Red line shows the solid position.

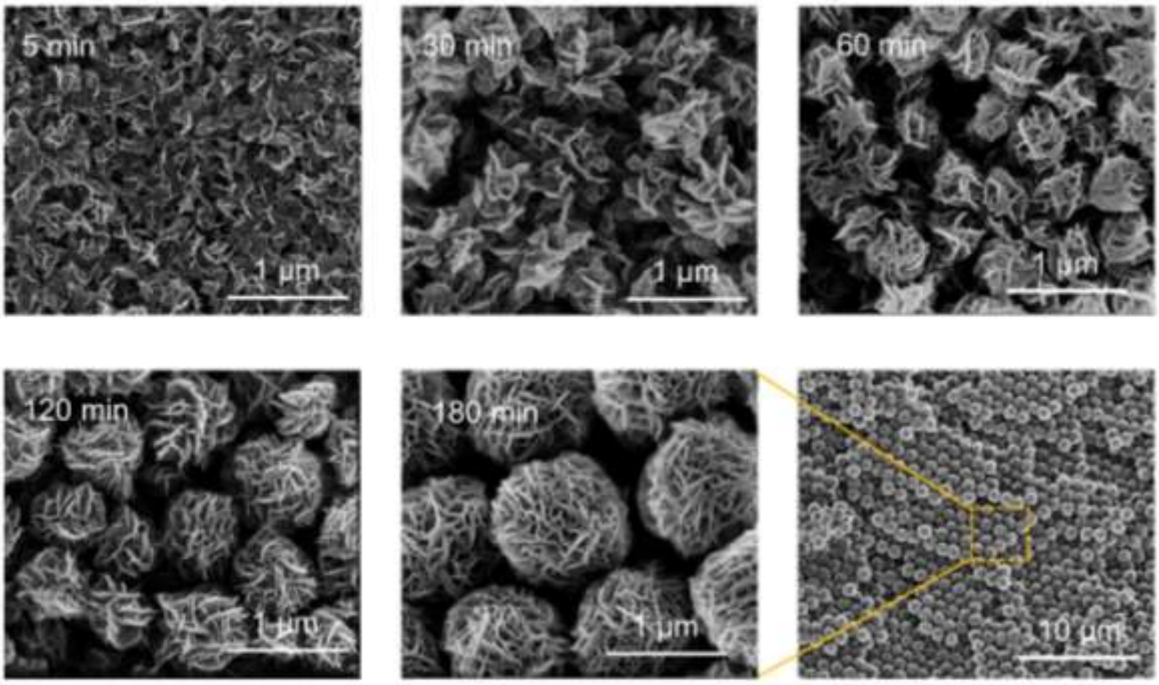

Figure S7. SEM images showing the PAN particle morphology at different stages of reaction (time of reaction) since its initation at 70°C.

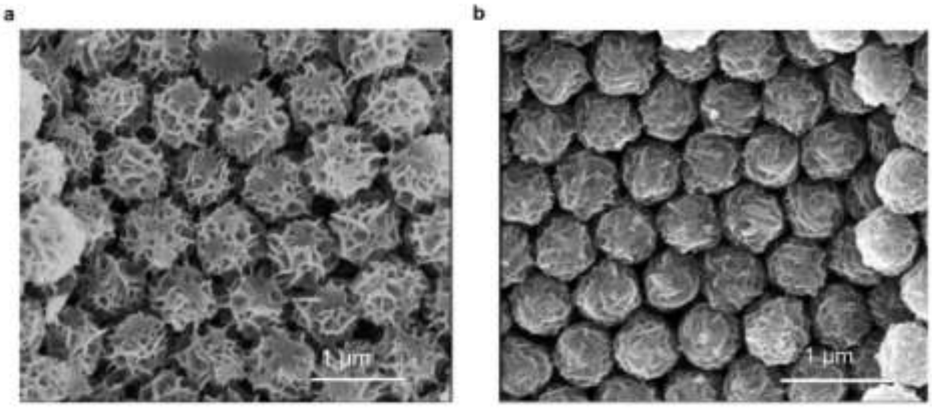

Figure S8. (a) A SEM image of an ACFP-SA synthesized in tetrahydrofuran (THF) as solvent. (b) A SEM image of an ACFP-SA synthesized in pyridine as solvent.

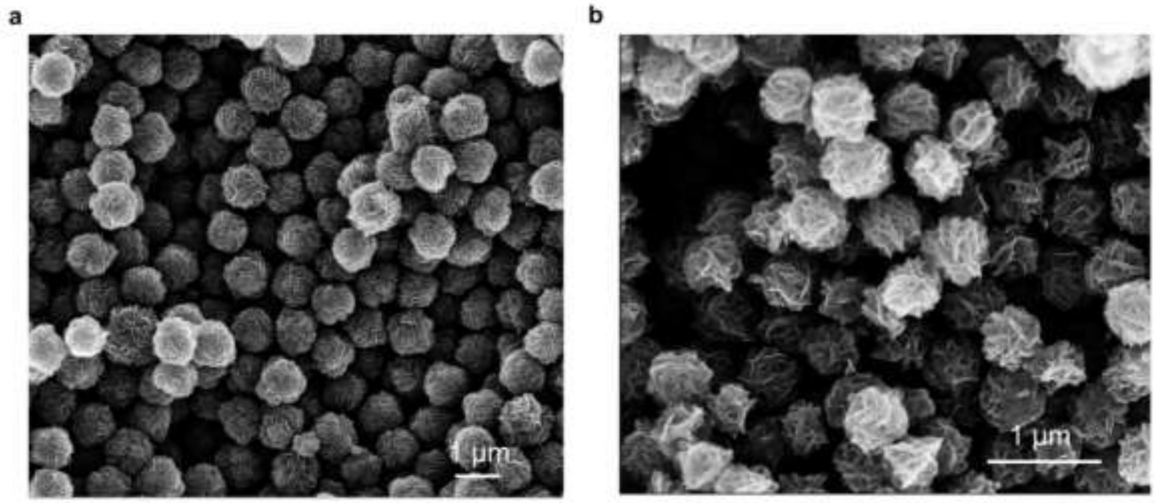

Figure S9. (a) A SEM image of an ACFP-VF (b) A SEM image of an ACFP-MC

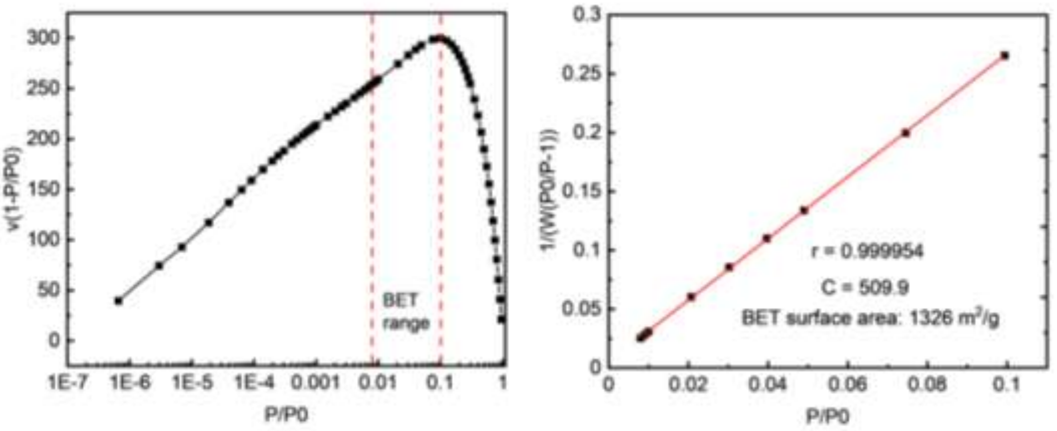

Figure S10. Selection of BET surface area calculation range (left) and its corresponding multi-point BET plot (right)

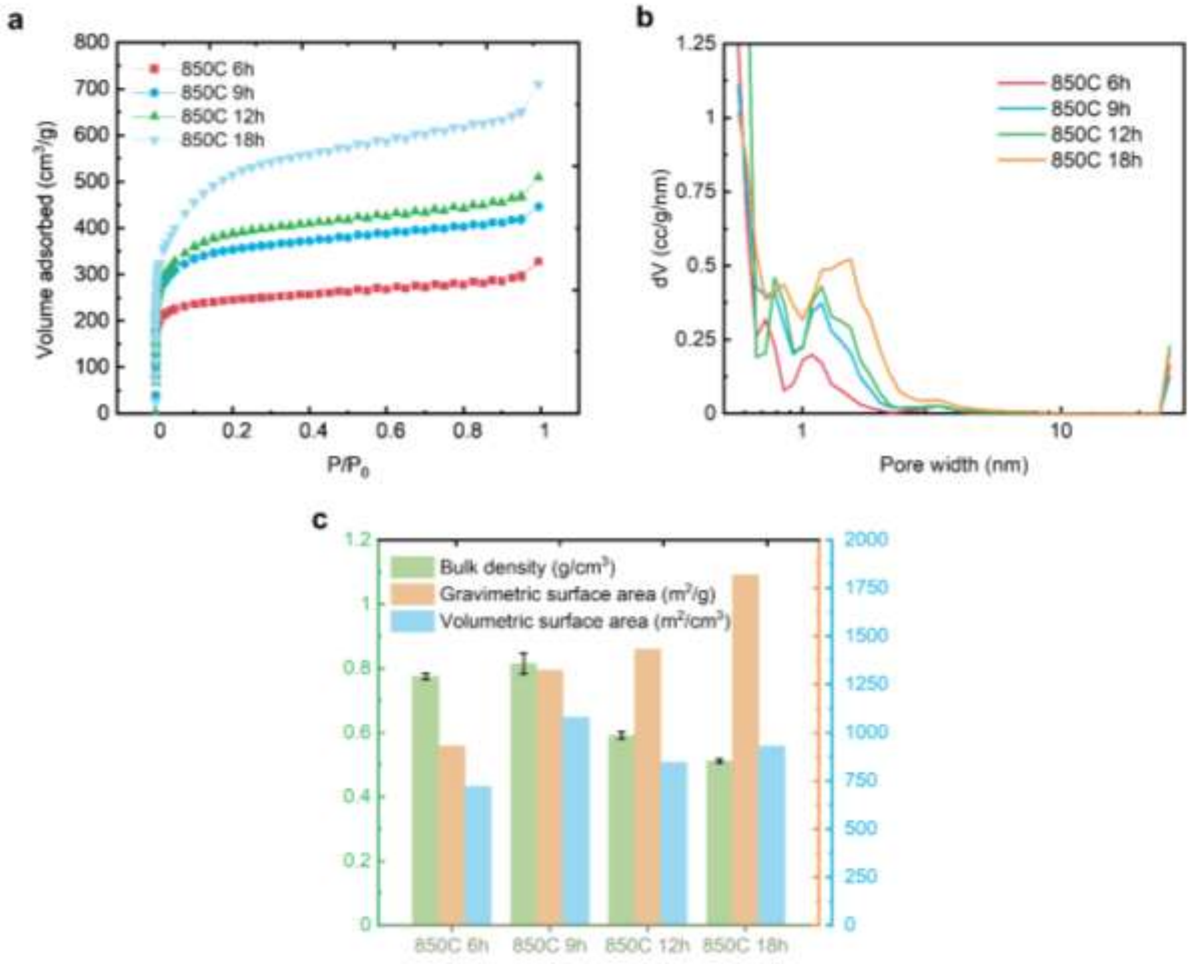

Figure S11. Effects of CO_2 activation time on the surface area and bulk density of ACFP-SA. (a) N_2 adsorption/desorption isotherms (b) Pore size distribution calculated based on QSDFT (c) Bulk densities and surface areas

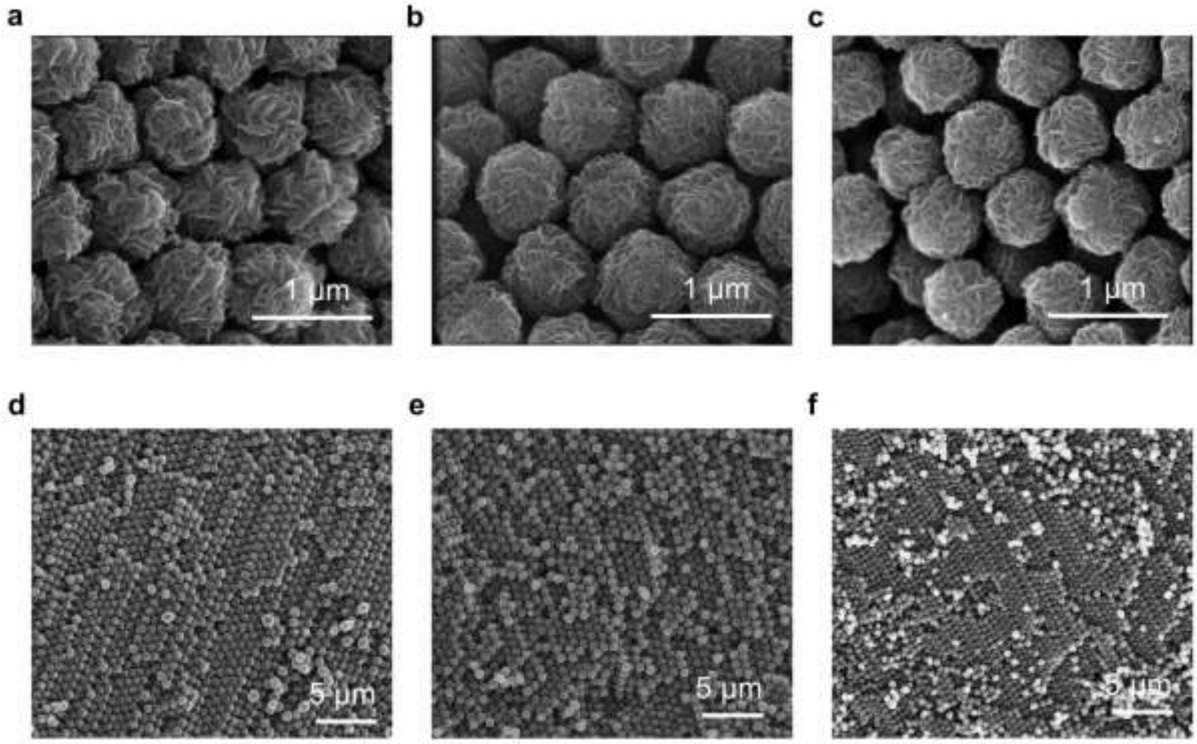

Figure S12. SEM images of ACFP-SA with different CO_2 activation times. (a, d) 850°C 6 h. (b, e) 850°C 12 h. (c, f) 850°C 18 h.

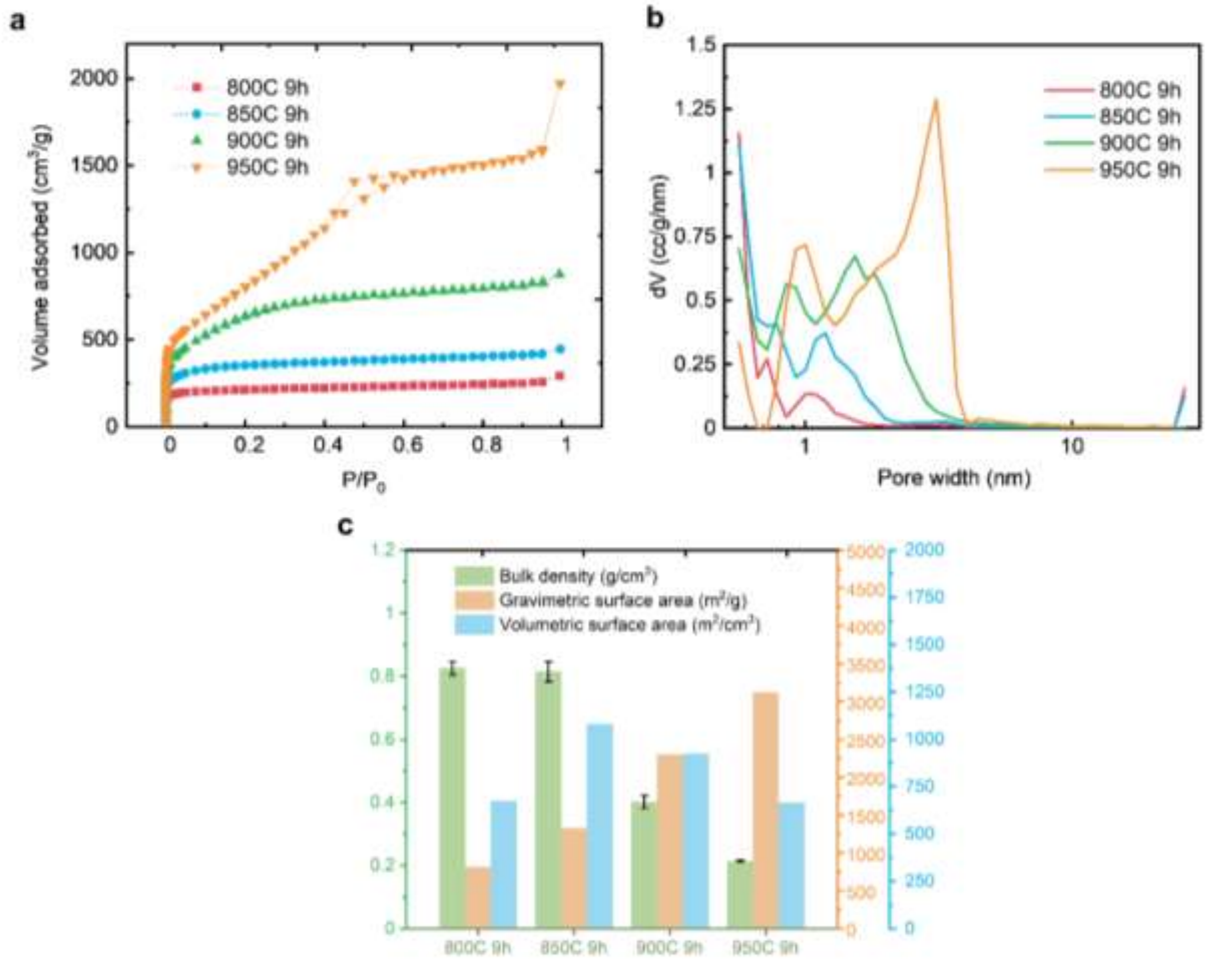

Figure S13. Effects of CO_2 activation temperature on the surface area and bulk density of ACFP-SA. (a) N_2 adsorption/desorption isotherms (b) Pore size distribution calculated based on QSDFT (c) Bulk densities and surface areas

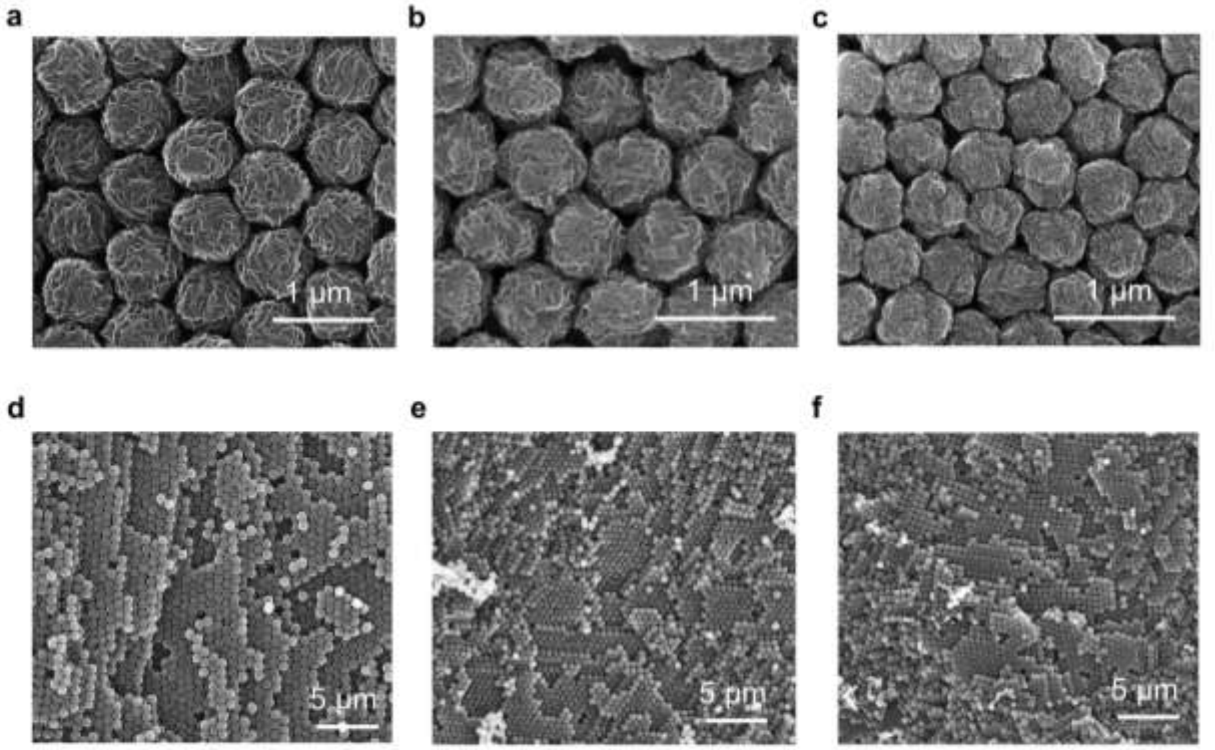

Figure S14. SEM images of ACFP-SA with different CO_2 activation temperatures. (a, d) 800°C 9 h. (b, e) 900°C 9 h. (c, f) 950°C 9 h.

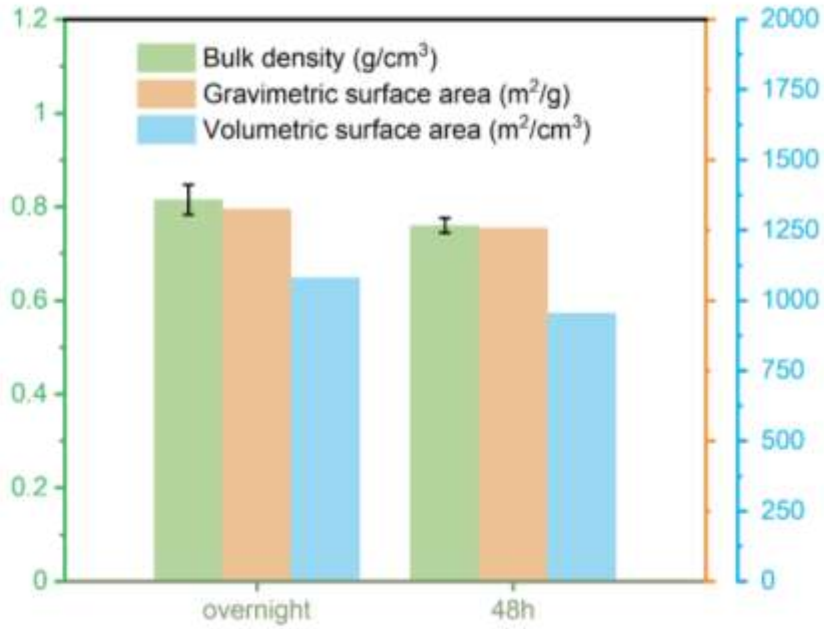

Figure S15. Effects of polymerization time on the surface area and bulk density of ACFP-SA.

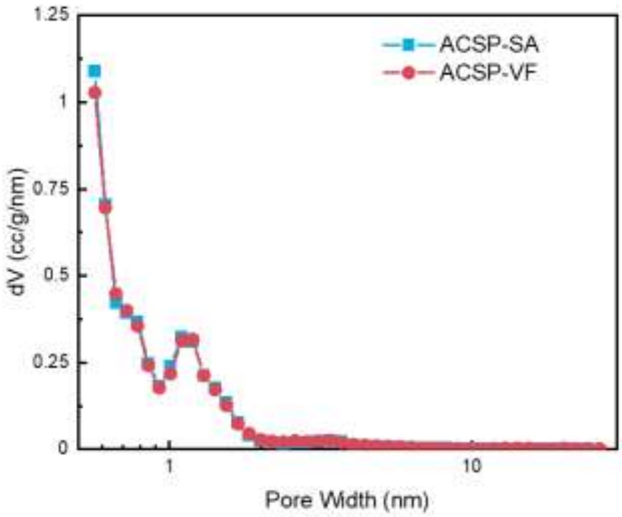

Figure S16. Pore size distribution of ACSP-SA and ACSP-VF calculated by QSDFT method

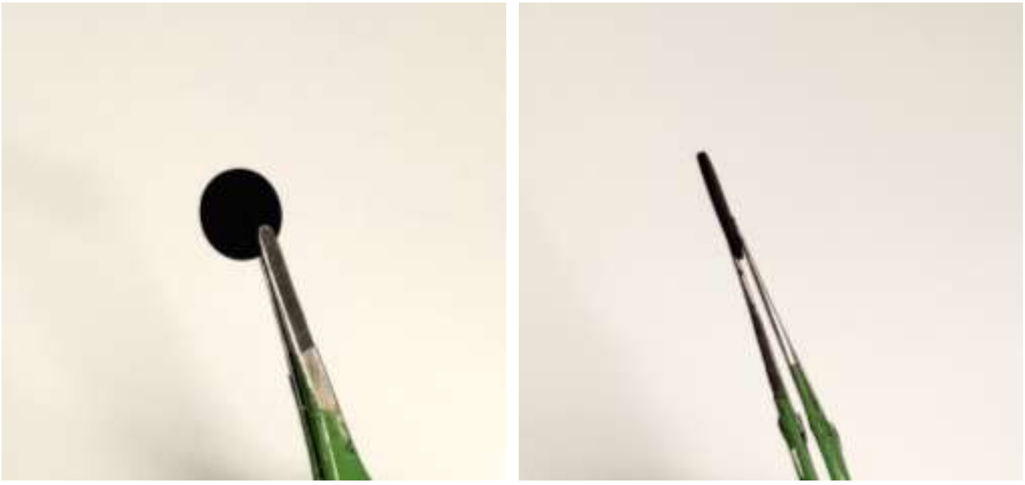

Figure S17. Freestanding thin sheets for supercapacitors

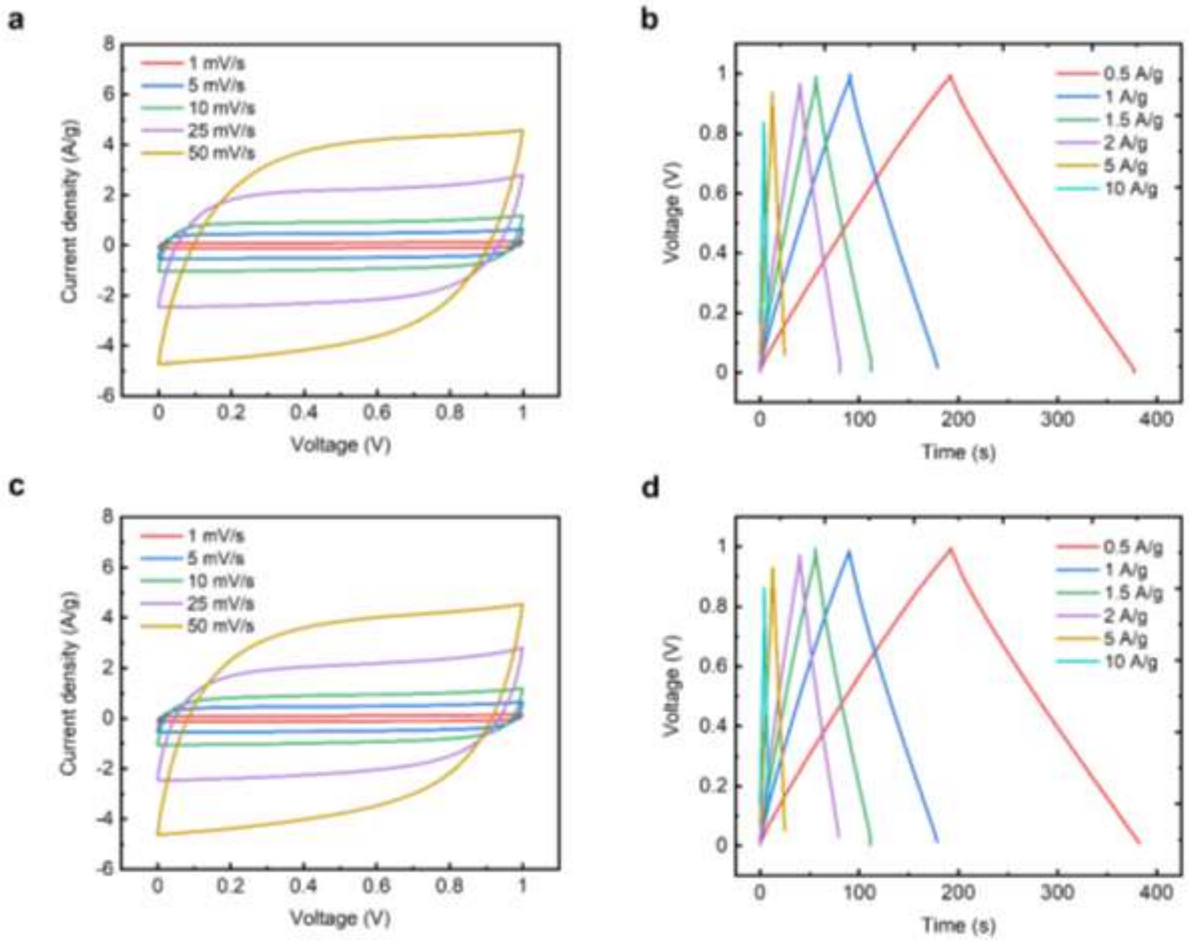

Figure S18. CV and GCD curves of ACFP-SA (a, b) and ACFP-VF (c, d)

Table S1. Gravimetric surface area, volume metric surface area, bulk density, and number of synthetic steps of different porous carbon materials

Type	Reference number	Gravimetric surface area (m^2/g)	Volumetric surface area (m^2/cm^3)	Bulk density (g/cm^3)	Number of synthetic steps
**Graphene-derived carbon**	7	390	421	1.08	17
	10	750	533	0.71	10
	11	830	590	0.71	8+
	15	1655	1258	0.76	10+
	17	400	600	1.5	10
	34	2927	1290*	0.44*	10+
	48	32	83	2.59	5
	51	720	763	1.06	8
	52	310	434	1.4	5+
	60	220	246	1.12	10+
**Polymer-derived carbon**	37	1586	1268	0.8	4+
	39	653	470	0.72	8+
	41	2186	498	0.23	10
	42	683	317	0.46	5
	45	889	258	0.29	5
	47	1872	468	0.25	9+
**Biomass-derived carbon**	33	1202	1010*	0.84*	8+
	38	1750	875	0.5	5
	43	715	369	0.52	7
**Carbide-derived carbon**	54	1500	1470	0.98	5+
	55	872	174	0.2	8+
	56	2300	256	0.11	7
	57	2933	774	0.26	10+
	58	1600	727	0.45	7+

	59	2420	1210*	0.50*	7+
	54&61	~1500	~765	0.51	7+
	61&62	~1500	~705	0.47	7+
**Silica-templated carbon**	32	2060	510*	0.25*	6+
	40	1540	260	0.17	8
	44	727	145	0.2	14
	49	688	716	1.04	10+
	50	1590	95	0.06	10
	53	1490	993*	0.67*	10+
**Zeolite-templated carbon**	63	1500	345	0.23	6+
	64	2710	510	0.19	7+
	64	1500	1340	0.89	7+
**Other carbon precursors**	13	1556	949	0.61	7+
	14	1000	570	0.57	3
	35	2299	804*	0.35*	9
	36	1044	438	0.42	12
	46	591	43	0.07	7

Numbers with “*” are calculated based on the total pore volume measurement by N_2 adsorption, which is overestimated according to our discussion. The actual bulk density of these materials is not reported. Numbers with “+” are synthetic processes that use commercial carbon precursors. We include three extra steps for those processes accounting for synthesis, carbonization, separation and mixing of carbon precursors.
